# Identification of Salivary Exosome-Derived miRNAs as Potential Biomarkers for Non-Invasive Diagnosis and Proactive Monitoring of Inflammatory Bowel Disease

**DOI:** 10.3390/ijms26167750

**Published:** 2025-08-11

**Authors:** Congyi Yang, Jingyi Chen, Yuzheng Zhao, Yalan Xu, Jushan Wu, Jun Xu, Feng Chen, Ning Chen

**Affiliations:** 1 Department of Gastroenterology, Peking University People’s Hospital, Beijing 100044, China; 2 Central Laboratory, Peking University School of Stomatology, Beijing 100081, China; 3 Clinical Center of Immune-Mediated Digestive Diseases, Peking University People’s Hospital, Beijing 100044, China

**Keywords:** salivary exosomes, inflammatory bowel disease, non-invasive biomarker, diagnosis and monitoring

## Abstract

Inflammatory bowel disease (IBD), a chronic inflammatory disorder with relapsing/remitting characteristics, lacks reliable non-invasive biomarkers for accurate diagnosis and longitudinal monitoring. This study explored salivary exosomal miRNAs as potential biomarkers to address this unmet clinical need. Using discovery (24 IBD patients [11 active, 13 remission] and 6 healthy controls [HCs]) and validation cohorts (102 IBD patients [53 active, 49 remission] and 18 HCs), we analyzed miRNA profiles via reverse transcription quantitative PCR (RT-qPCR). Receiver operating characteristic (ROC) curves evaluated diagnostic performance, with area under the curve (AUC) quantifying discriminatory capacity. Initial screening revealed 23 miRNAs significantly upregulated in IBD salivary exosomes. An 8-miRNA signature distinguished IBD patients from HCs in validation analyses, with five miRNAs (hsa-miR-1246, hsa-miR-142-3p, hsa-miR-16-5p, hsa-miR-301a-3p, and hsa-miR-4516) showing strong correlations with disease activity. The combination of hsa-miR-16-5p and hsa-miR-4516 achieved robust discrimination (AUC = 0.925 for IBD vs. HCs; AUC = 0.82 for active disease vs. remission). A composite model integrating all five miRNAs demonstrated superior performance (AUC = 1.00 for IBD/HC differentiation; AUC = 0.86 for disease activity assessment). These findings reveal dynamic associations between salivary exosomal miRNA signatures and IBD progression, underscoring their utility as non-invasive diagnostic tools. This approach enables serial sampling, enhances patient compliance, and provides actionable insights for personalized disease management, establishing salivary exosomal miRNAs as promising candidates for clinical translation in IBD care.

## 1. Introduction

Inflammatory bowel disease (IBD), primarily comprising ulcerative colitis (UC) and Crohn’s disease (CD), exhibit a pattern of relapsing and remitting inflammation in the intestinal mucosa, which leads to debilitating symptoms and drastically diminishes patients’ quality of life [1].

The diagnosis of IBD currently lacks a gold standard, primarily relying on invasive endoscopic procedures with histopathological confirmation [2]. These methods impose significant costs, procedural risks, and patient discomfort. Conventional serum biomarkers such as erythrocyte sedimentation rate (ESR) and C-reactive protein (CRP) not only require invasive blood sampling but also demonstrate limited diagnostic specificity [3]. Accurate longitudinal monitoring of intestinal inflammation remains a critical unmet need for optimizing IBD therapy [4]. Consequently, a pressing requirement exists for non-invasive and reliable markers that can facilitate diagnosis, monitor disease activity, and guide therapeutic interventions.

Liquid biopsy has emerged as a pivotal diagnostic tool in modern medicine [5,6]. Among biofluids, saliva offers advantages for biomarker research due to non-invasive sampling, molecular congruence with blood components, and technical feasibility [7]. Compared to blood sampling requiring venipuncture, saliva collection avoids procedural risks while improving patient compliance. Physiologically, the salivary gland/circulatory system connection ensures transfer of plasma-derived biomarkers (DNA, RNA, proteins, metabolites) into saliva. Additionally, saliva’s non-coagulating nature simplifies laboratory processing by avoiding anticoagulant requirements. These attributes enable salivary biomolecules to reflect pathological changes, positioning saliva as a non-invasive medium for disease screening, activity monitoring, and therapeutic response evaluation [8,9]. Current evidence demonstrates multidimensional molecular alterations in IBD patients’ saliva: reduced antioxidant enzyme activities (e.g., glutathione and catalase) with concurrent elevated lipid peroxidation in active Crohn’s disease [10], elevated proinflammatory cytokines (IL-1β, IL-6, TNF-α) correlating positively with disease activity [11], and distinct microRNA expression patterns specifically miR-101 upregulation in Crohn’s disease versus miR-21/miR-31/miR-142-3p upregulation coupled with miR-142-5p downregulation in ulcerative colitis [12,13]. However, whole saliva analysis faces inherent limitations, including susceptibility to environmental contaminants and endogenous components like enzymes that degrade RNA/proteins, compromising the detection of low-abundance biomarkers [14,15]. These challenges have spurred interest in salivary exosomes, which encapsulate biomolecules within lipid bilayers, thereby enhancing analytical stability and sensitivity [16,17].

Exosomes, nanosized vesicles approximately 30 to 150 nanometers in diameter, are abundantly found in numerous biological media, including saliva [18,19]. The wide array of molecular components they carry mirrors the condition of their source tissues, providing singular prospects for disease detection and evaluation [20,21]. Salivary exosomes, which encapsulate a diverse array of biomolecules including DNA, microRNAs(miRNAs), and proteins, have emerged as promising candidates for disease diagnosis [14]. Notably, the exosomal protein PSMA7 in saliva demonstrates diagnostic consistency for IBD [22]. The analysis of exosomal miRNAs as non-invasive biomarkers has attracted considerable attention due to their relatively high abundance within exosomes and the sensitivity of current detection technologies [23,24,25]. Recent studies indicate that salivary exosomal miRNAs serve as diagnostic biomarkers not only for oral pathologies (e.g., oral cancer, periodontitis, oral lichen planus) but also for systemic malignancies such as pancreatobiliary tract and lung cancers [8,26,27,28,29,30]. Although existing evidence demonstrates dysregulated exosomal miRNAs in IBD cohorts [31,32], systematic profiling of salivary exosomal miRNA signatures for non-invasive diagnosis remains unexplored.

In this study, we aimed to characterize salivary exosome-derived miRNA profiles in inflammatory bowel disease and establish a non-invasive diagnostic biomarker panel. Through comparative analysis of exosomal miRNA expression patterns between IBD patients and healthy controls (HC), we identified a five-miRNA signature (hsa-miR-1246, hsa-miR-142-3p, hsa-miR-16-5p, hsa-miR-301a-3p, and hsa-miR-4516). Validation studies demonstrated the clinical utility of this signature for both disease detection and activity monitoring, supporting its potential application in precision medicine approaches for IBD management.

## 2. Results

### 2.1. Identifying Expression Patterns of Salivary Exosomal miRNAs in IBD

**Figure 1:**
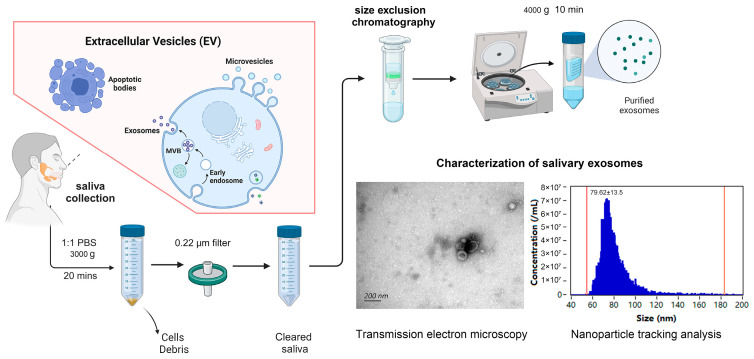
Isolation and characterization of salivary exosomes. TEM image showing cup-shaped morphology of salivary exosomes (scale bar = 200 nm). NTA plot demonstrating size distribution and purity of isolated particles (79.62 ± 13.5 nm). This figure was created in BioRender. Congyi, Y. https://BioRender.com/kob8qfx (accessed on 2 August 2025).

**Figure 2:**
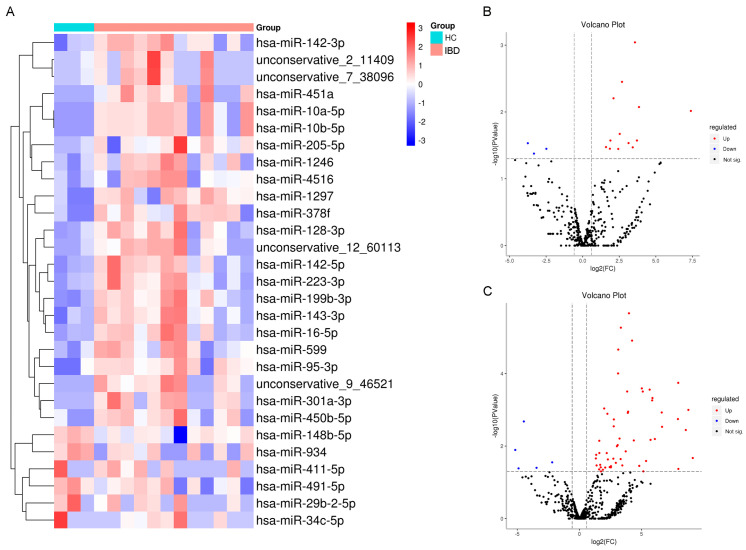
The levels of salivary exosomal miRNAs vary significantly between IBD patients and healthy individuals. (**A**) Heatmap of salivary exosomal miRNAs in IBD patients and HC. (**B**) The volcano plot reveals distinct differential expression patterns of miRNAs in salivary exosomes between patients with CD and HC. (**C**) The volcano plot reveals distinct differential expression patterns of miRNAs in salivary exosomes between patients with UC and HC. The red hue signifies an increase in expression, whereas the blue hue denotes a decrease. *p*-value < 0.05 and |FC| > 2. UC: Ulcerative colitis. CD: Crohn’s disease. HC: Healthy controls.

We initially quantified exosomal miRNA expression in salivary samples from 24 IBD patients stratified by clinical phase (active: n = 11; remission: n = 13) and 6 HC. The demographic and related data of the study subjects and the control group are summarized in Table S2. Nanoparticle tracking analysis (NTA) was employed to quantify the biophysical properties of salivary exosomes, revealing a mean particle size distribution of 79.62 ± 13.5 nm. Transmission electron microscopy (TEM) with uranyl acetate negative staining confirmed the characteristic cup-shaped morphology of exosomes, validating vesicle structural integrity (Figure 1). Additionally, exosomal characteristic markers (CD63/Tsg101) were identified using Western blot (Figure S1). This study is exploratory and aims to preliminarily screen miRNAs with potential associations; therefore, a raw *p*-value < 0.05 is temporarily adopted as the screening criterion to retain more candidate signals. We observed that 23 miRNAs were upregulated, as identified within salivary exosomes from the IBD group, while 6 miRNAs showed downregulation (Figure 2A). Subsequently, we conducted separate analyses for CD and UC specimens. In the analysis of the CD cohort, we identified 13 miRNAs that exhibited higher expression within salivary exosomes from individuals with CD, as opposed to those observed in healthy individuals. The elevated miRNAs include hsa-miR-451a, hsa-miR-378f, hsa-miR-4516, hsa-miR-205-5p, hsa-miR-599, hsa-miR-1297, hsa-miR-199b-3p, hsa-miR-92b-3p, hsa-miR-1246, hsa-miR-203a-3p, hsa-miR-143-3p, hsa-miR-223-3p, and hsa-miR-142-5p. Contrarily, the expression of hsa-miR-29b-2-5p, hsa-miR-34c-5p, and hsa-miR-590 in salivary exosomes from individuals with CD was notably diminished relative to that in healthy counterparts (Figure 2B). Moreover, our approach revealed a differential expression of 56 miRNAs between UC patients and HC. Specifically, 54 miRNAs demonstrated increased expression. Additionally, salivary exosomes from UC patients exhibited decreased levels of hsa-miR-34c-5p and hsa-miR-934, contrasting with those found in HC (Figure 2C).

### 2.2. Functional Analysis of Differentially Expressed miRNAs (DEMs) of Salivary Exosomes

**Figure 3:**
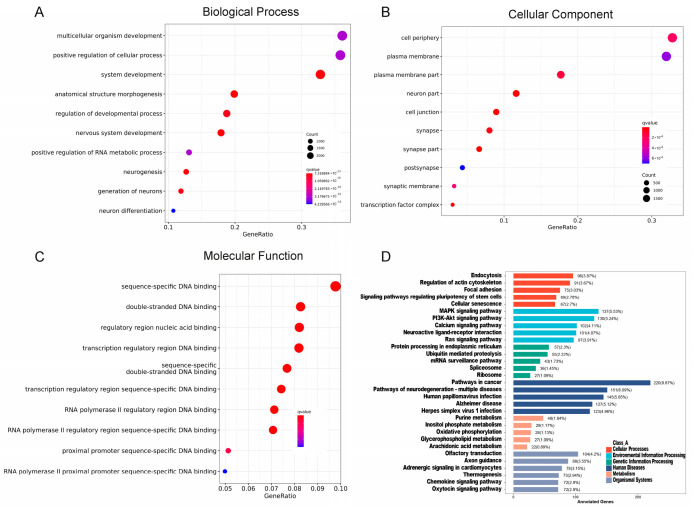
A targeted evaluation of the DEMs within the salivary exosome fractions distinguished between IBD and HC. A bubble chart depicts the enrichment of GO terms in Biological Processes (**A**), Cellular Components (**B**), and Molecular Functions (**C**). (**D**) KEGG pathways enriched and quantification of DEM target genes across various pathways.

**Figure 4:**
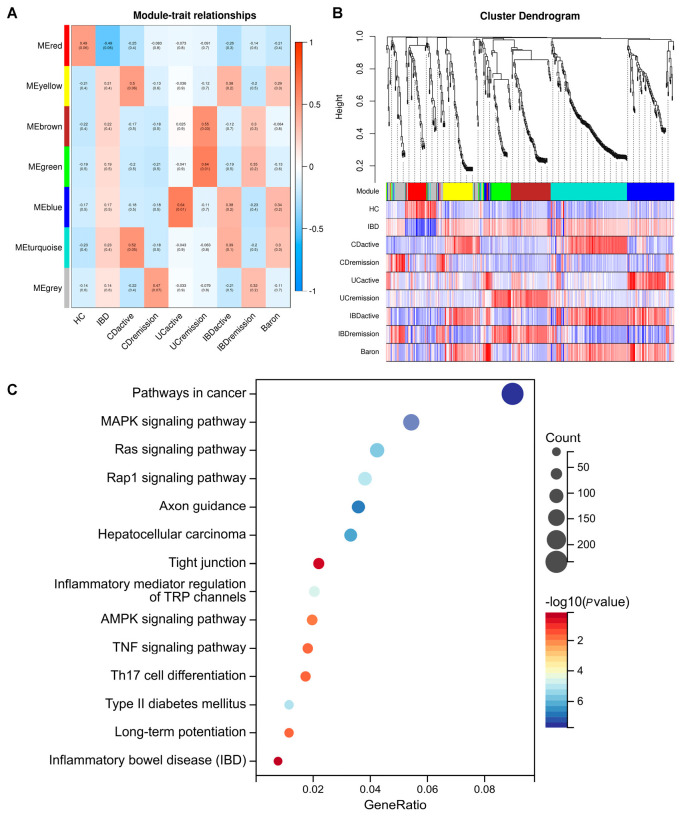
Identification of modules linked to clinical features of IBD using WGCNA. (**A**) Cluster dendrogram of co-expressed genes in IBD. (**B**) Heatmap of module–trait relationships in IBD. (**C**) KEGG pathways enriched and quantification of DEM target genes across various pathways.

To clarify the roles of distinct miRNAs present in the salivary exosomes of IBD patients compared to those in HC, a comparative analysis of their potential impact on protein-coding mRNAs was conducted. Table S3 lists the names and functional properties of these differentially expressed miRNAs. Enrichment analysis using GO and KEGG databases, along with gene databases for miRNA interactions, revealed that the differentially expressed miRNAs predominantly targeted genes implicated in multicellular organism development, tight junction formation, DNA binding, ubiquitin-mediated proteolysis, oxidative phosphorylation, and pathways associated with cancer (Figure 3). Through WGCNA, we identified genes closely related to the occurrence of IBD compared to HC, which were grouped into modules, each represented by a different color (Figure 4A,B). Taking into account the number of genes and the degree of correlation, we selected the active CD-related turquoise module and active UC-related blue module as the target modules. Subsequent KEGG enrichment analysis (Figure 4C) indicated that the differentially expressed genes within these modules were primarily enriched in pathways associated with inflammatory bowel diseases (Table S4), Th17 cell differentiation, and tight junctions.

### 2.3. Identification of the Salivary Exosomal miRNAs Signature Correlated with IBD Disease Activity

**Figure 5:**
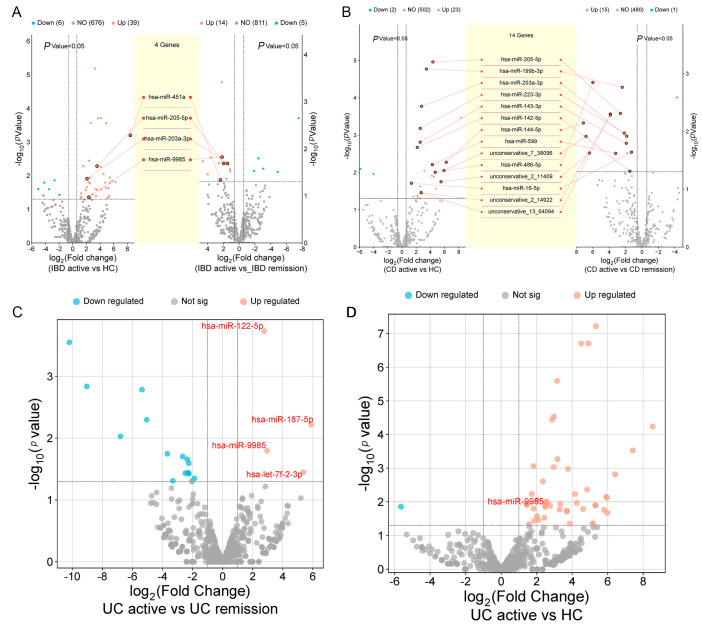
DEMs in salivary exosomes of IBD patients with differing disease activity. (**A**) A ternary volcano plot illustrates the overlap of DEMs between active IBD patients and HC, as well as between active IBD patients and those in remission. (**B**) A ternary volcano plot illustrates the DEMs between patients with CD and HC, and also between active CD individuals and those in remission. (**C**) A volcano plot illustrates the DEMs between patients with UC active and UC remission. (**D**) A volcano plot illustrates the DEMs between patients with UC active and HC. *p*-value < 0.05 and fold change > 2.

After identifying dysregulated miRNAs in salivary exosomes of IBD patients, we assessed their association with disease activity. Comparative analysis revealed that hsa-miR-203a-3p, hsa-miR-205-5p, hsa-miR-451a, and hsa-miR-9985 were consistently upregulated in both comparisons: active IBD vs. healthy controls and active IBD vs. remission-stage patients (Figure 5A).

In CD patients, 15 miRNAs exhibited heightened expression in salivary exosomes during the active phase of the disease, compared to levels observed in individuals in remission. Of particular note, the expression of 14 of these miRNAs in salivary exosomes was significantly higher in active CD patients than in both HC and IBD patients in remission (Figure 5B). In the subgroup analysis of the UC cohort, we analyzed miRNA expression in exosomes extracted from the saliva of UC patients during active episodes and periods of remission. This analysis revealed that 4 miRNAs (hsa-let-7f-2-3p, hsa-miR-122-5p, hsa-miR-187-5p, hsa-miR-9985) were markedly underexpressed in individuals with UC in remission compared to those in the active phase. We also noticed a significant increase in the expression levels of hsa-miR-9985 in patients with active UC compared to those in remission and compared to the HC group.

### 2.4. Correlation Between Salivary Exosomal miRNAs with Clinical Parameters

**Figure 6:**
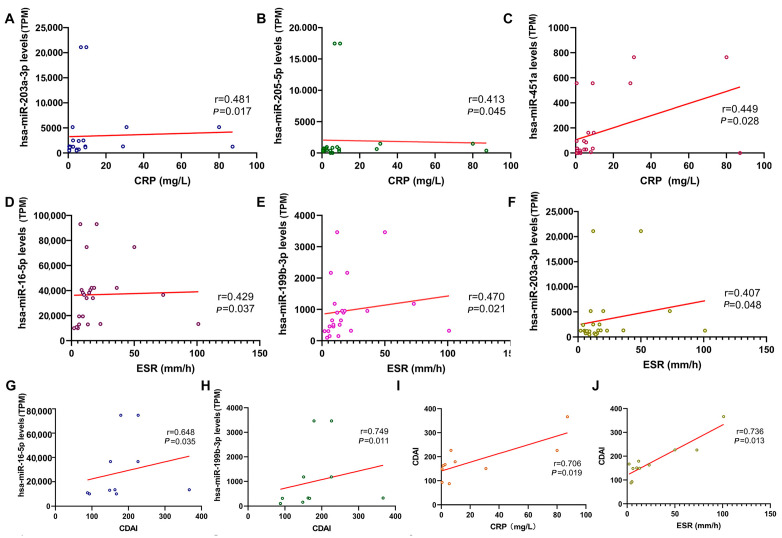
Relationship between miRNA expression profiles in salivary exosomes (quantified via miRNA-seq) and clinical parameters. (**A**–**C**): Correlation between salivary exosomal miRNA levels and CRP. (**D**–**F**): Correlation between salivary exosomal miRNA levels and ESR. (**G**–**J**): Correlation between CDAI and salivary exosomal miRNA levels (**G**,**H**), CRP (**I**), and ESR (**J**). Statistical analyses were performed using Spearman’s correlation test. Statistical significance was defined as *p* < 0.05.Transcripts Per Million (TPM).

CRP and ESR are commonly used clinical biomarkers for detecting inflammation. We performed correlation analyses to determine whether salivary exosomal miRNA levels (quantified via miRNA-seq) could reflect these inflammatory biomarkers and disease severity in IBD patients (Figure 6A–F). Among miRNAs differentially expressed in IBD patients compared to healthy controls (HC), the levels of hsa-miR-205-5p and hsa-miR-451a showed a notable correlation with CRP levels (*p* = 0.045, r = 0.413; *p* = 0.028, r = 0.449). Additionally, the levels of hsa-miR-16-5p, hsa-miR-199b-3p, and hsa-miR-203a-3p were significantly correlated with ESR levels (*p* = 0.037, r = 0.429; *p* = 0.021, r = 0.470). When analyzing microRNA expression changes specifically in IBD patients comparing the active phase to the remission phase, hsa-miR-203a-3p levels exhibited significant correlations with both CRP and ESR (*p* = 0.017, r = 0.481; *p* = 0.048, r = 0.407).

To assess their potential as biomarkers for disease activity, we correlated miRNA levels with clinical activity scores (Mayo score for UC, n = 13; CDAI for CD, n = 11). Hsa-miR-16-5p and hsa-miR-199b-3p levels significantly correlated with CDAI (*p* = 0.035, r = 0.648; *p* = 0.011, r = 0.749). The correlation coefficients for CRP vs. CDAI and ESR vs. CDAI were 0.706 and 0.736, respectively. Notably, the correlation between hsa-miR-199b-3p and CDAI (r = 0.749) was stronger than these established biomarker correlations (Figure 6G–J). This suggests that salivary exosome-derived miRNAs could serve as reliable indicators of CD activity.

### 2.5. Validation Analysis of Salivary Exosomal miRNAs in Independent Cohort

**Figure 7:**
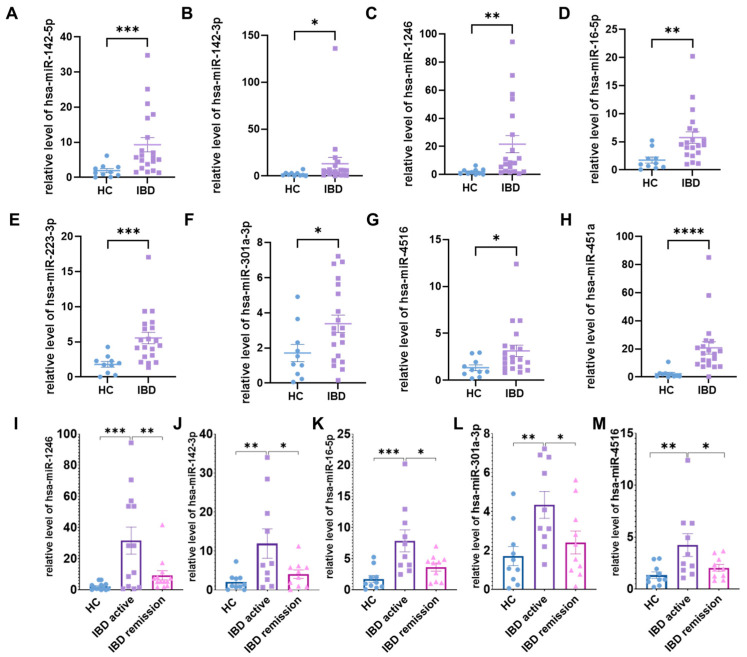
Validation Analysis of Salivary Exosomal miRNAs in IBD Patients and HC. (**A**–**H**) Salivary exosomal miRNA differed significantly between IBD patients and HC. (**I**–**M**) Comprehensive assessment of miRNA expression in salivary exosomes from IBD patients relative to objective disease manifestations. Active disease was defined by the presence of both clinical symptoms and endoscopic lesions. Remission was defined by the absence of clinical symptoms and normal colonoscopy findings. Data are presented as mean ± SEM. Data from RT-PCR CT values, corrected with reference gene U6 to calculate ΔCT (target-U6 CT). 2^(−ΔΔCT)^ method was used for relative quantification, with results shown as fold changes vs. control. * *p* < 0.05, ** *p* < 0.01, *** *p* < 0.001, **** *p* < 0.0001.

To validate the salivary exosomal miRNAs initially identified in the discovery cohort of IBD patients, we measured their expression levels using qRT-PCR in an independent validation cohort (n = 120). Table 1 details the clinical characteristics of the validation cohort. Among the 29 miRNAs with altered expression identified in the discovery cohort, 8 were successfully replicated in this validation cohort, accounting for approximately 28% of the candidate miRNAs. We observed that the levels of hsa-miR-1246, hsa-miR-142-3p, hsa-miR-142-5p, hsa-miR-16-5p, hsa-miR-223-3p, hsa-miR-301a-3p, hsa-miR-4516, and hsa-miR-451a were significantly elevated in IBD patients compared to HC (Figure 7A–H). Notably, the levels of hsa-miR-1246, hsa-miR-142-3p, hsa-miR-16-5p, hsa-miR-301a-3p, and hsa-miR-4516 exhibited a correlation with disease activity, showing an increase in the active state and a decrease in remission, approaching levels observed in healthy controls. (Figure 7I–M; Figure S2).

**Figure 8:**
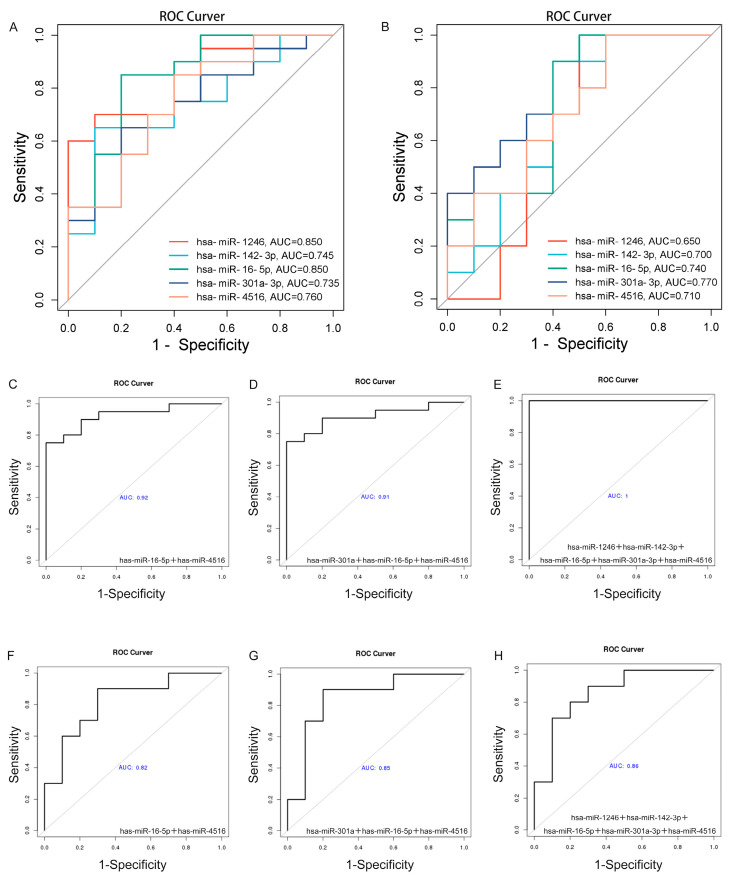
Validation of Salivary Exosomal miRNAs for Screening and Predicting IBD Activity. (**A**) Diagnostic biomarkers (IBD vs. healthy controls): ROC curves of miRNAs distinguishing IBD patients from healthy individuals. (**B**) Activity biomarkers (IBD active vs. remission): ROC curves reflecting disease activity. (**C**–**E**) Diagnostic biomarker panels for IBD screening. (**F**–**H**) Activity biomarker panels for monitoring disease progression. ROC curves were generated from the miRNA qRT-PCR validation cohort.

To evaluate the diagnostic potential of these miRNAs as biomarkers, we performed receiver operating characteristic (ROC) analysis and calculated the area under the curve (AUC). Specifically, hsa-miR-1246 and hsa-miR-16-5p exhibited AUCs of 0.85 each for distinguishing IBD patients from HC, while hsa-miR-142-3p, hsa-miR-301a-3p, and hsa-miR-4516 achieved AUCs of 0.745, 0.735, and 0.76, respectively (Figure 8A). Collectively, these five miRNAs showed promising AUC values for differentiating IBD patients from HC. Further ROC analysis comparing patients in the active phase to those in remission yielded AUC values of 0.65 for hsa-miR-1246 and 0.74 for hsa-miR-16-5p. Additionally, hsa-miR-142-3p, hsa-miR-301a-3p, and hsa-miR-4516 demonstrated AUCs of 0.7, 0.77, and 0.71, respectively (Figure 8B).

Additionally, we utilized a logistic regression model that incorporated the candidate miRNAs, demonstrating enhanced diagnostic accuracy and activity assessment for IBD compared to their individual effects. Specifically, the joint detection of hsa-miR-16-5p and hsa-miR-4516 demonstrated an AUC of 0.925 for distinguishing IBD patients from HC (Figure 8C), and an AUC of 0.82 in differentiating active disease from remission (Figure 8F). Moreover, adding hsa-miR-301a to this combination resulted in an AUC of 0.91 for differentiating IBD patients from controls (Figure 8D) and an AUC of 0.85 for distinguishing active disease from remission (Figure 8G). However, the addition of a fourth candidate miRNA to the model did not further improve the ability to distinguish active disease from remission (Figure S3). The final predictive model, which incorporated all five miRNAs, achieved an AUC of 1.00 for distinguishing IBD patients from HC (Figure 8E) and an AUC of 0.86 for differentiating active disease from remission (Figure 8H).

## 3. Discussion

The diagnosis of IBD remains clinically challenging, primarily due to the absence of both an established gold standard and validated disease-specific biomarkers [33,34]. There is an urgent need to identify reliable, non-invasive markers to facilitate both diagnosis and longitudinal monitoring of IBD. This study demonstrates that salivary exosomal miRNAs may serve as indicators of IBD status, offering novel possibilities for the diagnosis and management of IBD.

Exosomes, membrane-bound nanoscale extracellular vesicles, contain bioactive substances reflecting their cell of origin and exhibit biological functions comparable to those of their parent cells—specifically, functions related to intercellular signaling, including the transfer of regulatory miRNAs and cytokines that modulate recipient cell activities [18]. Secreted by nearly all cell types, they persist in diverse bodily fluids [16]. Research indicates their potential for tracking IBD progression [35]. Exosomes isolated from the intestinal lumen of IBD patients showed significantly elevated concentrations of inflammatory mediators (IL-6, IL-8, IL-10, TNF-α) compared to healthy individuals [36]. Shao et al. identified 292 differentially expressed proteins in serum exosomes from IBD patients versus controls, including significantly upregulated pregnancy zone protein—an immune suppressor [37]. Liu et al. reported elevated expression of long non-coding RNA NEAT1 in serum and tissues of IBD patients [38]. Collectively, these and other studies establish exosomes’ ubiquitous presence in biological fluids as a foundation for biomarker development [39,40]. The exosomal lipid bilayer protects internal RNAs/proteins, enhancing circulatory stability beyond other biomolecules [18]. Their tissue-specific trafficking further confers unique advantages as diagnostic and monitoring biomarkers. Consequently, investigating exosomes as diagnostic and monitoring tools for IBD progression represents a priority research frontier, potentially enabling innovative approaches to IBD management.

As a systemic immune-mediated disorder, IBD primarily affects the intestines but frequently presents with extraintestinal manifestations across multiple organ systems [41,42]. The oral cavity is commonly involved, with manifestations often mirroring intestinal disease activity—waxing and waning with inflammation control [43]. Recent IBD research has shifted focus to the oral microbiome. Our prior studies demonstrated reduced diversity in the salivary microbiome of IBD patients, accompanied by compositional shifts and altered microbial abundance [44]. These findings have been corroborated by subsequent investigations [45]. Thus, saliva from IBD patients likely contains more comprehensive biomarkers reflecting intestinal inflammation.

Compared to other biofluids, saliva offers advantages including ease of collection, cost-effectiveness, non-invasiveness, and high patient compliance. However, comprehensive analysis of whole saliva is limited by contamination susceptibility and protein denaturation [46,47]. Salivary exosome analysis effectively overcomes these limitations. Our previous work identified >2000 distinct proteins in salivary exosomes from IBD patients, with 8 exhibiting significant upregulation [22]. In this study, we focus on salivary exosomes and their miRNA content as promising non-invasive biomarkers for IBD diagnosis and disease activity assessment. This study enrolled patients with confirmed IBD diagnoses, stratified by disease activity and subtype. However, the absence of treatment-based stratification may introduce confounding factors influencing miRNA profiles. Future investigations in treatment-naïve cohorts are needed to validate this miRNA panel’s diagnostic accuracy, to define and validate diagnostic thresholds, and determine the false positive (FP) and false negative (FN) rates.

As a type of non-coding RNA, miRNAs have been linked to colitis in various studies [12,48]. However, free miRNA in body fluids is inherently unstable and can be easily degraded by various RNases in vivo, which greatly limits its potential as a direct biomarker. Exosomes offer a protective mechanism for miRNA. Owing to their lipid bilayer architecture, exosomes are adept at encapsulating and preserving the integrity of encapsulated miRNA, shielding them from external disruptions such as RNases, extreme pH conditions, and temperature fluctuations. Consequently, miRNA encapsulated within exosomes demonstrates increased stability, positioning it as an attractive prospect for potential biomarker applications [49,50,51].

A study has revealed that serum levels of miR-223 and miR-4516 are pronouncedly downregulated in UC individuals during remission compared to those with active disease [52]. Another study has indicated increased levels of miR-16 and miR-223 in fecal samples from individuals with CD and UC, which are higher than those in control samples [53]. In this research, we observed that levels of has-miR-223-3p, has-miR-4516, and hsa-miR-16-5p were markedly higher in the salivary exosomes of individuals with IBD compared to the healthy group. Two primary factors may account for these inconsistencies. First, divergent biological matrices (serum, feces, saliva) yield distinct miRNA expression profiles, as selective miRNA partitioning may reflect localized IBD pathophysiology. Second, variations in experimental methodologies—including RNA extraction, normalization strategies, and targeted miRNA isoforms—likely contribute to such discrepancies. Notably, miR-4516 and hsa-miR-16-5p demonstrated a substantial direct association with the severity of disease activity in IBD patients. In IL-10-/-mice with spontaneous colitis, miR-142-3p and miR-142-5p were significantly increased in colonic mucosa [54]. Upregulation of miR-143 and miR-301a expression has been documented in the inflamed intestinal lining of IBD sufferers [55,56,57]. In line with these findings, our research observed elevated concentrations of hsa-miR-142-3p, hsa-miR-142-5p, and hsa-miR-301a in the salivary exosomes of IBD patients compared to the HC group. Liu et al. elucidated the function of miR-301a, identifying its capacity to exacerbate colonic mucosal inflammation, impair immune responses, and enhance progression of colitis-related cancer via suppression of SNIP1 and BTG1, while this effect is reversible with anti-miR-301a [56,57]. These studies suggest that miRNAs might hold relevance for both detection and treatment protocols in IBD. Our findings indicate that has-miR-301a in salivary exosomes serves not only as a predictive marker for IBD but also effectively distinguishes between active and remission phases. The expression of miR-1246 is notably increased in serum and fecal specimens from IBD patients [58,59]. Similarly, we observed a rise in the concentration of has-miR-1246 within salivary exosomes of IBD patients, with a more pronounced increase in those with active IBD. Interestingly, miR-1246 has subsequently been identified to alleviate colonic inflammation in an experimental colitis model [60]. These findings elucidate that the interplay between IBD and miRNAs remains intricate. A similar contradiction occurred in studies of miR-451a. Although it has been associated with alleviation of various inflammatory disorders [61,62], a higher concentration of miR-451a is now linked to a heightened likelihood of post-surgical recurrence in CD [63]. Consistent with previous studies, we observed elevated levels of hsa-miR-451a in salivary exosomes derived from IBD patients compared to levels in healthy individuals.

Salivary exosomes harboring miRNAs present a novel perspective for disease diagnosis, mirroring the pathological states of IBD patients. The elevation of these miRNAs may be associated with immune system abnormalities in active IBD patients. Since these miRNAs regulate gene expression, their abnormal expression may lead to excessive production of inflammation-related genes, thereby causing immune dysregulation and exacerbating the inflammatory response. Although the precise function of these miRNAs in IBD pathogenesis remains unclear, their elevated expression may serve as biomarkers for IBD and contribute to disease diagnosis and activity assessment. However, it should be noted that the limited scale of the discovery cohort necessitates multicenter validation to assess treatment-related confounders before clinical application for primary diagnosis. Future studies should focus on identifying subtype-specific molecular markers differentiating UC and CD. Furthermore, ongoing study and validation of miRNA signatures in salivary exosomes may open avenues for identifying innovative therapeutic targets and interventions targeting IBD.

## 4. Materials and Methods

### 4.1. IBD Diagnosis and Activity

Diagnosis for IBD and subtype was confirmed by clinical manifestation, endoscopy, and histology. Disease activity was determined by clinical manifestation and endoscopic appearance. CD activity was evaluated with CDAI, including height, weight, hematocrit, symptoms, complications, etc. Ant CDEI was introduced to assist in the assessment of endoscopy activity. Therefore, the active CD was determined by CDAI ≥ 150 or CDEI ≥ 5. For patients divided into UC subtypes, the modified Mayo disease activity index, containing bowel frequency, rectal hemorrhaging, assessment by a doctor, and endoscopy findings, was used. A Mayo score ≥ 3 was determined as active UC.

### 4.2. Cohort for Extraction of Salivary Exosomal miRNAs and Clinical Information

The samples were allocated into two distinct cohorts: the discovery and validation cohorts. The discovery cohort encompassed 24 IBD patients (11CD, 13UC) along with 6 healthy controls (HC) devoid of history pertaining to IBD or any other immune-mediated inflammatory diseases. Comprehensive clinical data are presented in Table S2. The validation cohort, comprising 120 participants, consisted of 102 IBD patients (42CD, 60UC) and 18 HC. Within this cohort, there were 53 active IBD patients (16CD, 37UC) and 49 in remission (26CD, 23UC). Basic information, clinical course, disease activity, and treatment are shown in Table 1. Serological testing for inflammatory biomarkers, including CRP and ESR, was conducted concurrently with saliva collection.

### 4.3. Western Blot

The concentration of exosomal proteins was determined using a BCA protein quantification kit (Thermo, Waltham, MA, USA). Based on the quantification results, the loading amount of exosomes (20 µg) was calculated, and 5× SDS buffer was added in proportion; after vortexing to mix thoroughly, the mixture was denatured in a 95 °C water bath for 5 min before protein electrophoresis was performed. After electrophoresis, the separating gel was removed, and the target protein in the gel was electrotransferred to a PVDF membrane. Following blocking with 5% BSA blocking solution, the membrane was incubated with primary antibodies against exosomal positive protein markers, including CD63 (abcam, 1:1000) and TSG101 (abcam, 1:1000), and then with secondary antibodies; subsequently, development, fixation, and exposure were carried out.

### 4.4. Extraction and Classification of Salivary Exosomes

Saliva samples were collected from all patients and controls after resting for 15 min in a quiet room. Participants remained seated in a relaxed state with the tongue tip pressed against the palate and head naturally lowered, allowing unstimulated whole saliva to passively drain into a 50mL centrifuge tube until approximately 5 mL was collected. Verbal communication was prohibited during the procedure, and samples were immediately placed on ice for preservation post-collection. Subsequently, these samples were centrifuged at 3000× *g* for 20 min at 4 °C to pelletize insoluble fragments, cellular material, and cells. The resultant precipitate was removed, and the supernatant was filtered through a 0.22 micron membrane (Millipore, Darmstadt, Germany) for further purification. The specimen after filtering was then loaded onto a size exclusion chromatography (SEC) column (Echobiotech, Beijing, China), which had been pre-rinsed. Following the complete loading of the sample onto the SEC column, an initial elution with 1.5 mL of PBS was performed. A subsequent elution with 2.5 mL of PBS yielded the fraction enriched with exosomes, which was collected for further processing. The exosome-enriched fraction was centrifuged at 4000× *g* for a duration of 10 min using a 100 kDa Amicon Ultra centrifugal filter (Millipore, Darmstadt, Germany) to concentrate and purify the exosomes. The nanoparticle tracing analysis approach (Flow NanoAnalyzer) was employed to characterize the purified exosomes, and the structure of the exosomes was assessed utilizing transmission electron microscopy (TEM).

### 4.5. Exosomal RNA Isolation and RNA Analyses

A starting volume of 2–5 mL of saliva samples was used for EV isolation. For the extraction of total RNA, the miRNeasy Mini Kit (Qiagen, Venlo, The Netherlands) was employed, following the manufacturer’s protocol. The RNA Nano 6000 Assay Kit (Agilent Technologies, Santa Clara, CA, USA) was utilized to ascertain the RNA yield and purity.

### 4.6. The Preparation and Sequencing of the Library

Small RNAs were processed with 1–5 ng input following the QIAseq miRNA Library Kit protocol. Libraries were purified using AMPure XP and assessed via Agilent Bioanalyzer 2100 and qPCR. Sequencing clusters were generated with the acBot system and the TruSeq PE Cluster Kit v3-cBot-HS. Libraries were sequenced on the Illumina HiSeq platform, generating paired-end reads of 150 bp in length, with an average of 25.50 million raw reads per sample.

### 4.7. Profiling miRNA Expression and Comparative Analysis

Raw sequencing reads were processed to remove low-quality sequences (Phred score < 20), eliminate reads with ≥10% undetermined bases (N), and discard reads lacking the 3’ adapter. Following adapter trimming, sequences shorter than 15 nt or longer than 35 nt were excluded. Using Bowtie, cleaned read segments were mapped against various databases like Silva, GtRNAdb, Rfam, and Repbase to filter out non-coding RNAs and repetitive elements. The filtered reads were then used to identify known miRNAs from miRbase and predict new miRNAs by aligning with the human genome reference (GRCh38). Leveraging Unique Molecular Identifiers (UMIs) incorporated during library preparation using the QsRNA-seq method [64], PCR amplification bias was corrected to ensure precise miRNA quantification. The miRNA expression was normalized by calculating Transcripts Per Million (TPM) from the aligned read counts.

### 4.8. Gene Ontology (GO) and Kyoto Encyclopedia of Genes and Genomes (KEGG) Pathway Enrichment Analysis

MultiMiR (v2.3) was employed to predict miRNA target genes, with selection based on endorsement from more than three databases or experimental validation. GO and KEGG pathway enrichments were analyzed using topGO in R and KOBAS in Python (v3.10), respectively [65].

### 4.9. Weighted Gene Co-Expression Network Analysis (WGCNA)

The WGCNA workflow was executed with the following parameters: (1) Genes with FPKM (Fragments Per Kilobase of transcript per Million mapped reads) ≥ 1 were retained; (2) Genes demonstrating a coefficient of variation (CV) ≥ 0.5 were selected for analysis; (3) An unsigned weighted co-expression network was constructed; (4) Dynamic modules were defined via hierarchical clustering using the Dynamic Hybrid Tree Cut algorithm; (5) A soft threshold power of 6 was applied to optimize signal detection; (6) Modules containing fewer than 30 genes were excluded; (7) Modules with similarity ≥ 0.4997 were merged during the final consolidation step.

### 4.10. Real-Time Quantitative PCR (qRT-PCR)

The miRcute Plus miRNA First-Strand cDNA Synthesis Kit (Tiangen, Beijing, China) facilitated the conversion of total RNA into cDNA. Gene expression was measured with TaqMan^®^ qPCR, using a 2 µL aliquot of the resulting cDNA for each reaction. The specific primers and probes are listed in Table S1.

### 4.11. Statistical Analysis

Statistical analyses were performed using GraphPad Prism (v8.0) and SPSS (v23.0). Continuous variables are expressed as mean ± standard error of the mean (SEM). Comparisons between two groups with normally distributed data were conducted using an independent samples t-test. For comparisons among multiple groups with normal distribution, one-way analysis of variance (ANOVA) was employed, followed by the least significant difference (LSD) post hoc test for pairwise comparisons when homogeneity of variance was assumed. Nonnormally distributed data were analyzed using nonparametric tests: the Mann–Whitney U test for two groups and the Kruskal–Wallis test with Dunn correction for multiple groups. Pearson correlation analysis was applied to assess relationships between salivary exosomal miRNA expression levels and inflammatory markers. A statistically significant result was defined by a *p*-value below 0.05.

### 4.12. Data and Code Availability

The raw sequence data reported in this paper have been deposited in the Genome Sequence Archive (GSA-Human) of the National Genomics Data Center, China National Center for Bioinformation/Beijing Institute of Genomics, Chinese Academy of Sciences, with the accession number HRA007892. It can be accessed upon request through the link https://ngdc.cncb.ac.cn/gsa-human/ (accessed on 2 August 2025). The raw sequence data reported in this paper will be shared upon request to the lead corresponding author (chenning79@bjmu.edu.cn). Any additional information required to reanalyze the data reported in this paper is available from the lead contact upon request.

## 5. Conclusions

In conclusion, our research indicates that exosomal miRNAs in saliva hold substantial promise as biomarkers for detecting and monitoring disease activity in IBD. Compared to traditional diagnostic methods, this approach provides greater convenience and non-invasiveness, thereby improving patient adherence. Additionally, it enables repeated sampling and analysis over short intervals, which is advantageous for timely diagnosis and tracking disease fluctuations in IBD patients.

## Figures and Tables

**Table 1:** Cohort for validation of salivary exosome miRNAs and Clinical information.

Description of Validation Cohort			Total	UC	CD	Control
Total	Patients (no. %)		120, 100%	60, 50%	42, 35%	18, 15%
	Sex (f/m)		49/71	28/32	12/30	9/9
	Age (mean + SD)		40 ± 16.5	46.4 ± 14.6	32.8 ± 16.2	
	Clinical Course (year, mean + SD)		6.1 ± 5.7	6.6 ± 5.9	5.5 ± 5.5	
Active	Patients (no. %)		53, 100%	37, 69.8%	16, 30.2%	
	Sex (f/m)		23/30	16/21	7/9	
	Age (mean + SD)		40 ± 15	44.3 ± 14.0	30.8 ± 14.9	
	Clinical Course (year, mean + SD)		5.6 ± 5.2	6.3 ± 5.7	4.2 ± 3.6	
	Mayo (mean + SD)			4.5 ± 2.0		
	CDAI (mean + SD)				177.2 ± 69.8	
	Serum (mean + SD)	Patients (no. %)	45, 100%	29, 64.4%	16, 35.6%	
		CRP (mg/L)	19.0 ± 29.5	14.3 ± 23.5	27.6 ± 37.4	
		Patients (no. %)	43, 100%	27, 60.0%	16, 40.0%	
		ESR (mm/h)	25.6 ± 20.6	28.7 ± 23.3	20.5 ± 14.1	
Remission	Patients (no. %)		49, 100%	23, 46.9%	26, 53.1%	
	Sex (f/m)		17/32	12/11	5/21	
	Age (mean + SD)		41.4 ± 17.9	49.7 ± 15.2	34.1 ± 17.1	
	Clinical Course (year, mean + SD)		6.7 ± 6.2	7.1 ± 6.3	6.4 ± 6.3	
	Mayo (mean + SD)			0.4 ± 0.8		
	CDAI (mean + SD)				40.1 ± 28.4	
	Serum (mean + SD)	Patients (no. %)	41, 100%	15, 36.6%	26, 63.4%	
		CRP (mg/L)	1.4 ± 1.3	1.2 ± 1.2	1.5 ± 1.3	
		Patients (no. %)	39, 100%	14, 35.9%	25, 64.1%	
		ESR (mm/h)	9.5 ± 9.4	11.8 ± 11.4	8.2 ± 8.1	
Treatment	Corticosteroids (no. %)		5, 100%	3, 60%	2, 40%	
	5-ASA (no. %)		52, 100%	40, 76.9%	12, 23.1%	
	Immunosuppressive treatment (no. %)		6, 100%	5, 83.3%	1, 16.7%	
	TNFα Inhibitor (no. %)		31, 100%	13, 41.9%	18, 58.1%	
	IL-12/23 Inhibitor (no. %)		14, 100%	1, 7.1%	13, 92.9%	
	Integrin Inhibitor (no. %)		16, 100%	10, 62.5%	6, 37.5%	
	Surgery (no. %)		0%	0%	0%	

## Data Availability

The raw sequence data reported in this paper will be shared upon request to the lead corresponding author (chenning79@bjmu.edu.cn). This paper does not report original code. Any additional information required to reanalyze the data reported in this paper is available from the lead contact upon request.

## Supplementary Materials

The following supporting information can be downloaded at: https://www.mdpi.com/article/10.3390/ijms26167750/s1.

## Abbreviations

The following abbreviations are used in this manuscript:

IBD	Inflammatory bowel diseases
CD	Crohn’s disease
UC	Ulcerative colitis
HC	Healthy control
MiRNAs	MicroRNAs
CRP	C-reactive protein
ESR	Erythrocyte sedimentation rate
NTA	Nanoparticle tracking analysis
TEM	Transmission electron microscopy
GO	Gene ontology
KEGG	Kyoto encyclopedia of genes and genomes
WGCNA	Weighted Gene Co-expression Network Analysis
DEMs	Differentially expressed miRNAs
SEC	Size exclusion chromatography
qRT-PCR	Real-Time Quantitative PCR
ROC	Receiver operating characteristic
AUC	Area under the curve
