# Supplementary material for: Identification of Salivary Exosome-Derived miRNAs as Potential Biomarkers for Non-Invasive Diagnosis and Proactive Monitoring of Inflammatory Bowel Disease
Source: Int J Mol Sci. 2025 Aug 11;26(16):7750. doi: 10.3390/ijms26167750 (PMC12386383; doi:10.3390/ijms26167750)
Supplement: Supplementary file 1 [file ijms-26-07750-s001.zip › ijms-3796106-supplementary.pdf]

Supplemental Figures and Figure Legends

Primer name	Primer sequence (5′-3′)
JWFFXTY-R	GTGCAGGGTCCGAGGT
JWFTY-PROBE	CAGAGCCACCTGGGCAATTT
U6-RT	AACGCTTCACGAATTTGCGT
U6-S	CTCGCTTCGGCAGCACA
U6-A	AACGCTTCACGAATTTGCGT
U6probe	AGAAGATTAGCATGGCCCCTGCGCA
hsa-miR-1246-RT	CAGTGCAGGGTCCGAGGTCAGAGCCACCTGGGCAATTTTTTTTTTTCCTGCT
hsa-miR-1246-F	ACCGACCGAATGGATTTTTGG
hsa-miR-142-3p-RT	CAGTGCAGGGTCCGAGGTCAGAGCCACCTGGGCAATTTTTTTTTTTCCATA
hsa-miR-142-3p-F	ACGACGTGTAGTGTTTCCTACTT
hsa-miR-142-5p-RT	CAGTGCAGGGTCCGAGGTCAGAGCCACCTGGGCAATTTTTTTTTTAGTAGT
hsa-miR-142-5p-F	CCACCCACCATAAAGTAGAAAGC
hsa-miR-16-5p-RT	CAGTGCAGGGTCCGAGGTCAGAGCCACCTGGGCAATTTTTTTTTTCGCCAA
hsa-miR-16-5p-F	CCAGCCTAGCAGCACGTAAATA
hsa-miR-223-3p-RT	CAGTGCAGGGTCCGAGGTCAGAGCCACCTGGGCAATTTTTTTTTTTGGGGT
hsa-miR-223-3p-F	CGACGAGTGTCAGTTTGTCAAAT
hsa-miR-301a-3p-RT	CAGTGCAGGGTCCGAGGTCAGAGCCACCTGGGCAATTTTTTTTTTGCTTTG
hsa-miR-301a-3p-F	ACCGACCAGTGCAATAGTATTGT
hsa-miR-4516-RT	CAGTGCAGGGTCCGAGGTCAGAGCCACCTGGGCAATTTTTTTTTTGCCCCG
hsa-miR-4516-F	GGGAGGAGGGAGAAGGGT
hsa-miR-451a-RT	CAGTGCAGGGTCCGAGGTCAGAGCCACCTGGGCAATTTTTTTTTTAACTCA
hsa-miR-451a-F	CACCACCAAACCGTTACCATTAC

Table S1: Primer sequence of target miRNAs in RT-qPCR.

Description of Study Cohort			Total	UC	CD	HC
Total	Patients (no. %)		30, 100%	13, 43.3%	11, 36.7%	6, 20%
	Sex (f/m)		8/22	3/8	3/8	2/4
	Age (mean + SD)		36.7±14.7	47.3±12.7	28.0±12.8	29.7±6.28
	Clinical course (year)		3.6±4.1	3.6±4.4	3.7±3.9	
Active	Patients (no. %)		13, 100%	7, 53.8%	6, 46.2%	
	Sex (f/m)		3 / 10	1 / 6	2 / 4	
	Age (mean + SD)		38.9±15.4	43.1±15.4	34.0±15.1	
	Clinical course (year)		2.9±2.6	2.4±2.4	3.5±2.9	
	Activity score	CDAI			218.7±79.1	
		Mayo		4.3±2.6		
	Serum (mean + SD)	CRP (mg/L)	38.9±15.4	8.4±9.5	36.0±38.3	
		ESR (mm/h)	30.1±28.3	17.4±8.9	44.8±36.7	
Remission	Patients (no. %)		11	6, 54.5%	5, 45.5%	
	Sex (f/m)		3/8	2/4	1/4	
	Age (mean + SD)		37.9±16.8	52.2±4.9	20.8±1.8	
	Clinical course (year)		4.4±5.4	4.9±6.1	3.9±5.2	
	Activity score	CDAI			123.3±30.3	
		Mayo		0.5±0.8		
	Serum (mean + SD)	CRP (mg/L)	2.5±2.8	3.0±3.3	1.9±2.2	
		ESR (mm/h)	8.6±5.2	10.8±5.3	6.0±4.2	
Treatment	5-ASA (no. %)		5, 100%	5, 100%	0	
	Azathioprine (no. %)		0	0	0	
	TNFα Inhibitor (no. %)		10, 100%	4, 40%	6, 60%	
	IL-12/23 Inhibitor (no. %)		7, 100%	3, 42.9%	4, 57.1%	
	Integrin Inhibitor (no. %)		4, 100%	4, 100%	0	
	Surgery (no. %)		2, 100%	1, 50%	1, 50%	

Table S2: Cohort for extraction of salivary exosomal miRNAs and Clinical information. Disease Activity: Active CD was defined by CDAI≥150. Active UC was defined by Mayo≥3. UC: Ulcerative colitis. CD: Crohn’s disease. HC: Healthy controls.

MiRNA	Function	Reference (PMID)
Upregulated		
miR-10a	Suppress IL-12/23, inhibits DC andTh1/Th17 cell inflammation responses	25281418
miR-10b	Impairs intestinal barrier function	36343490
miR-1246	Suppress Nfat5 restoring Th17/Treg balance	34907166
miR-128-3p	Inactivate NF-κB pathway	34042286
miR-1297	Suppress IL-6, TNFα	38345447
miR-142-3p	Unknown	
miR-142-5p	Regulate SOCS1 and upregulate IL-6/8	31949659
miR-143-3p	Inhibit autophagy, increase inflammatory responses	29562274
miR-16-5p	Upregulate IFN-γ, IL-8, IL-1β and TNF-α	33182065
miR-199b-3p	Inhibit Nrf2, alleviate acute injury	34490833
miR-205-5p	Target XBP1, alleviate periodontitis;<br>Target MDM2, alleviate inflammatory response in rheumatoid arthritis	35234168
miR-233-3p	Promote cell apoptosis and inflammation	31210320
miR-301a-3p	Promote Th17 cell differentiation, promote metastatic and invasive ability in colorectal cancer	26338824
miR-378f	Unknown	
miR-450b-5p	Unknown	
miR-4516	Unknown	
miR-451a	Promote macrophages polarization of M1 to M2, inhibit inflammation	35395782
miR-599	Promote cell proliferation, inhibit cell apoptosis, inflammation response and oxidative stress	36156291
miR-95-3p	Unknown	
Downregulated		
miR-148b-5p	Unknown	
miR-29b-2-5p	Unknown	
miR-34c-5p	Inhibit ZO-1 and occludin	28153728
miR-491-5p	Unknown	
miR-934	Unknown	

Table S3: Data on the miRNAs that exhibit differential expression in salivary exosomes among IBD patients and HC.

Gene	Related MiRNAs
STAT1	hsa-miR-128-3p
TGFB2	hsa-miR-1306-5p
IL12RB1	hsa-miR-149-5p
FOXP3	hsa-miR-4516/hsa-miR-149-5p
RELA	hsa-miR-185-5p/hsa-miR-744-5p
IL17A	hsa-miR-24-3p/hsa-miR-6134
HLA-DRB5	hsa-miR-320c
IFNGR2	hsa-miR-378a-3p/hsa-miR-378a-3p
MAF	hsa-miR-10394-5p/hsa-miR-1306-5p/hsa-miR-339-3p/hsa-miR-423-5p/hsa-miR-4508/hsa-miR-4516/hsa-miR-7114-3p/hsa-miR-744-5p/unconservative_22_90333 hsa-miR-4446-3p
IL2RG	hsa-miR-423-5p
NFATC1	hsa-miR-4508/hsa-miR-7704//hsa-miR-6803-3p
IL12RB2	hsa-miR-4508//hsa-miR-4687-3p/unconservative_2_14922
TBX21	hsa-miR-4508/unconservative_22_90333/hsa-miR-6803-3p
SMAD3	hsa-miR-4508/hsa-miR-744-5p/hsa-miR-7704/unconservative_22_90333
IL12A	hsa-miR-4508/hsa-miR-744-5p
TGFB3	hsa-miR-4516
HLA-DQB1	hsa-miR-4687-3p/hsa-miR-542-5p
HLA-DRA	hsa-miR-4687-3p
TGFB1	hsa-miR-4787-5p/hsa-miR-6134/hsa-miR-744-5p/unconservative_22_90333/hsa-let-7g-3p/hsa-miR-6803-3p

Table S4: IBD-related Genes and Their Upstream MicroRNAs.

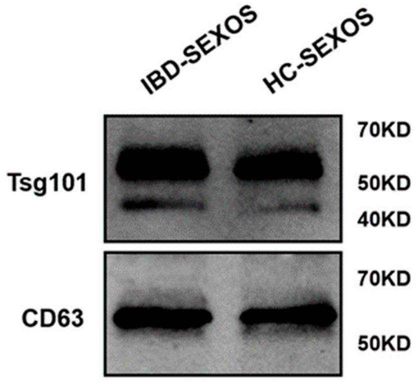

Figure S1: Western blot analysis of exosomal markers in isolated vesicles.
Representative Western blot images demonstrating the presence of exosomal markers Tsg101 and CD63 in exosomes isolated from saliva samples of inflammatory bowel disease (IBD) patients (IBD-SEXOs) and healthy controls (HC-SEXOs).

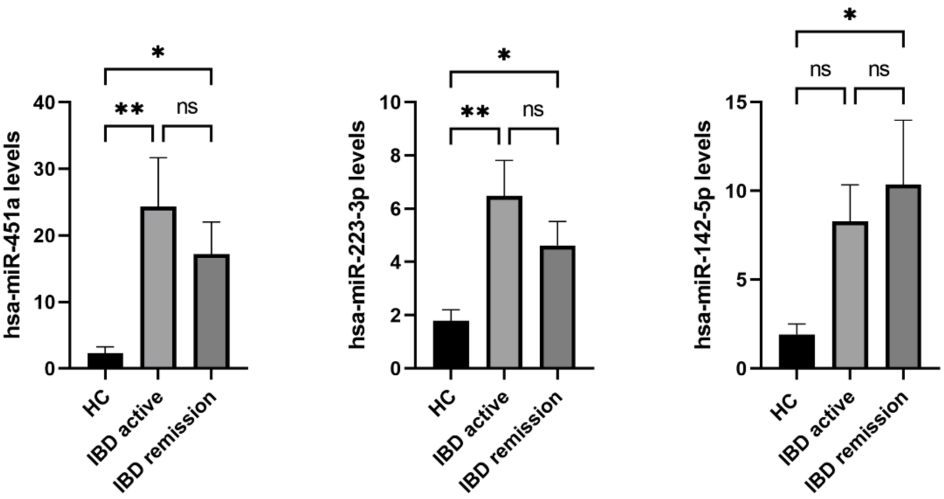

Figure S2: MiRNAs of salivary exosomes validation analysis in IBD patients based on objective disease activity. Active disease is defined by both clinical symptoms and active disease in colonoscopy. Remission is defined by both clinical symptoms and remission in colonoscopy. Data are represented as mean values ± SEM. Normalization was performed using U6 for equal volume of salivary exosomes. *P < 0.05, **P < 0.01, ***P < 0.001.

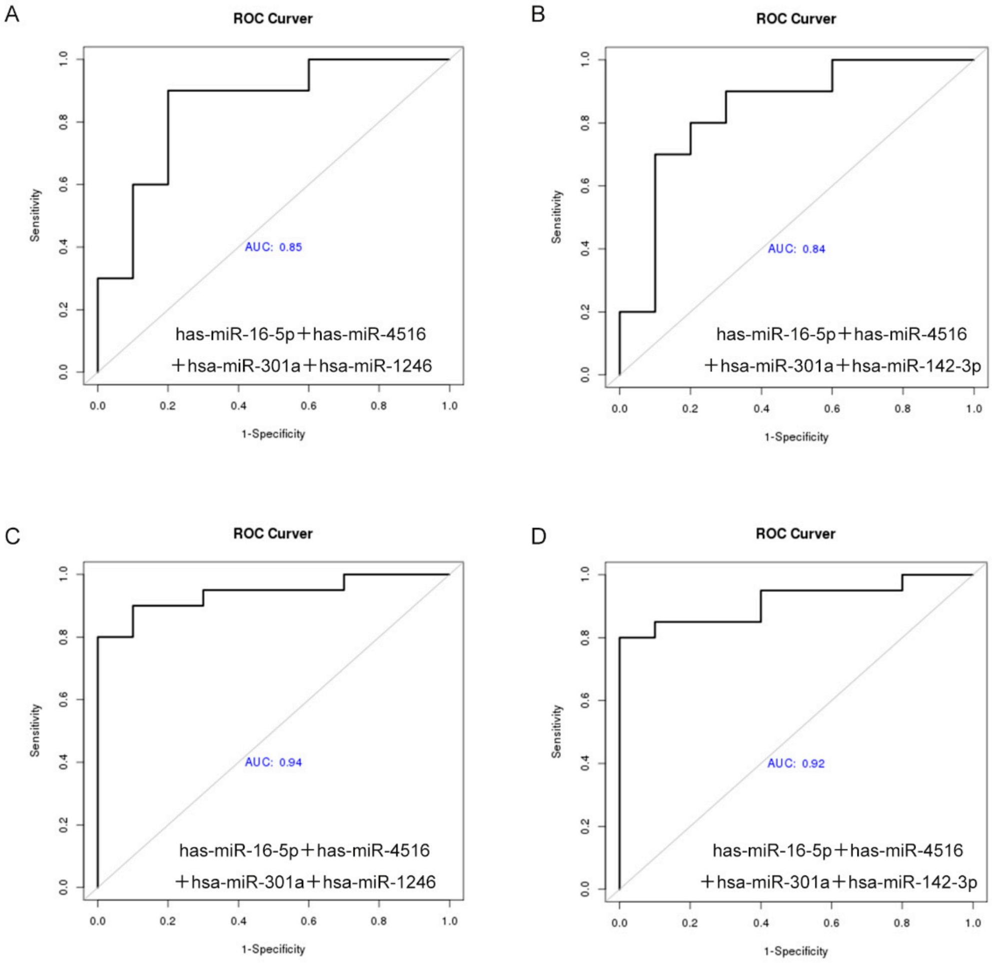

Figure S3: Performance of salivary exosomal miRNAs panels as biomarkers of IBD. Verification of panel 1 (A, hsa-miR-16-5p + hsa-miR-4516+hsa-miR-301a-3p + hsa-miR-1246), panel 2 (B, hsa-miR-16-5p + hsa-miR-4516+hsa-miR-301a-3p + hsa-miR-142-3P) as biomarkers of IBD. Performance of salivary exosomes miRNA panels as biomarkers for disease activity in IBD, verification of panel 1 (C), panel 2 (D). The ROC curves of validation dataset (miRNA RT-qPCR).
